# Interactome Mapping Reveals Important Pathways in Skeletal Muscle Development of Pigs

**DOI:** 10.3390/ijms151221788

**Published:** 2014-11-26

**Authors:** Jianhua Cao, Tinghua Huang, Xinyun Li, Shuhong Zhao

**Affiliations:** Key Laboratory of Agricultural Animal Genetics, Breeding and Reproduction of Ministry of Education of China, College of Animal Science and Technology, Huazhong Agricultural University, 1 Shizishan St., Wuhan 430070, China; E-Mails: jhcao@mail.hzau.edu.cn (J.C.); thua45@126.com (T.H.); hzaulxy@163.com (X.L.)

**Keywords:** pig, skeletal muscle, gene ontology, interactome

## Abstract

The regulatory relationship and connectivity among genes involved in myogenesis and hypertrophy of skeletal muscle in pigs still remain large challenges. Presentation of gene interactions is a potential way to understand the mechanisms of developmental events in skeletal muscle. In this study, genome-wide transcripts and miRNA profiling was determined for Landrace pigs at four time points using microarray chips. A comprehensive method integrating gene ontology annotation and interactome network mapping was conducted to analyze the biological patterns and interaction modules of muscle development events based on differentially expressed genes and miRNAs. Our results showed that in total 484 genes and 34 miRNAs were detected for the duration from embryonic stage to adult in pigs, which composed two linear expression patterns with consensus changes. Moreover, the gene ontology analysis also disclosed that there were three typical biological events *i.e.*, microstructure assembly of sarcomere at early embryonic stage, myofibril formation at later embryonic stage and function establishments of myoblast cells at postnatal stage. The interactome mappings of different time points also found the down-regulated trend of gene expression existed across the whole duration, which brought a possibility to introduce the myogenesis related miRNAs into the interactome regulatory networks of skeletal muscle in pigs.

## 1. Introduction

Muscle growth and development is of central importance for porcine carcass mass weight and quality. Skeletal muscle cells arise from embryonic mesoderm during embryonic development [1,2]. Multiple regulatory factors, including those of transcriptional factors and cellular signaling molecules, play a critical role in the control of muscle proliferation and differentiation [3,4,5]. The molecular basis underlying myogenesis and its regulatory networks remain a major challenge. With the development of detection technologies of gene expression such as microarray, next generation sequencing (NGS) such as RNA-Seq and real-time quantitative PCR, it is easier to obtain genome-wide data on gene expression. However, to decipher the biological meanings behind the expression data remains a difficult task. There were several studies on the development of skeletal muscle in pigs [6,7], in which the primary coverage focused on exploring the differentially expressed genes (DEGs) at different periods and few studies investigated the biological meaning and the relationships of DEGs.

The previous studies showed that the non-coding RNA, especially microRNAs (miRNAs), are widely considered as a type of regulator, associated with muscle growth and development, and play an important role in various regulatory mechanisms and interaction networks [8]. The muscle specifically expressed or highly enriched miRNAs, such as *miR-1*, *miR-133*, *miR-206* and *miR-208*, which are responsible for the transcriptional networks involving *SRF*, *MyoD*, *MEF2* and *myocardin*, and play central regulatory roles in myoblast proliferation and differentiation during myogenesis [8,9,10]. Aberrant miRNA expression or differentially expressed miRNAs (DEMs) have been observed during muscular dystrophy diseases [11]. Together, these studies indicated that miRNAs could be previously unrecognized key regulators of muscle cell proliferation and differentiation. Advances in understanding miRNA roles in skeletal muscles, combined with the accomplishment of the pig genome, have begun to reveal the miRNAs mediating molecular mechanisms of skeletal muscle in pigs.

It is generally acknowledged that there are two distinctive periods in the development of skeletal muscle of pigs, the prenatal period composed of early, middle and late embryonic stages and postnatal period from neonatal to adult [12]. Also, the number of muscle fibers was determined at the embryonic period, in which the primary fibers were formed at early stage and second fibers around the primary fibers were formed from middle to late stage. After birth, the number of muscle fibers was no longer increasing and developed the hypertrophy processes responding to nutrition status like amino acids, and there was no more myogenesis except for repair of muscle injury [13,14].

Although, recent studies involving skeletal muscle development in pigs largely converged functional characterization and regulatory networks based on RNA-Seq or microarray data, the core questions of the relationship and biological events behind the genes or miRNAs remain to be investigated [15,16]. Moreover, identifying the key nodes of networks remained difficult in these studies.

In the present study, we undertook the chip-based expression profiling for the DEGs and DEMs at four time points of skeletal muscle in pigs. The gene ontology annotation (GOA) analysis combined with interactome networks mapping was conducted on the DEGs to build the interaction networks for the four time points of skeletal muscle development in pigs. Our results disclosed that 484 DEGs and 34 DEMs constituted two linear expression patters (LEPs) responsible for the biological events in the development of skeletal muscles. Moreover, the interaction maps were created based on the DEGs and their GOA items, which revealed more detailed relationship and key nodes about the DEGs. Thus, on the basis of the interaction maps about porcine skeletal muscle, this work provided a new insight into understanding the interactions or modulation mechanisms of the development of skeletal muscle in pigs. Also, the study is likely to shed light on genes and gene networks that during 4 time points from embryo to adult can be involved to influence growth and development of porcine skeletal muscle.

## 2. Results and Discussion

### 2.1. Profiling of the DEGs and DEMs

**Figure 1:**
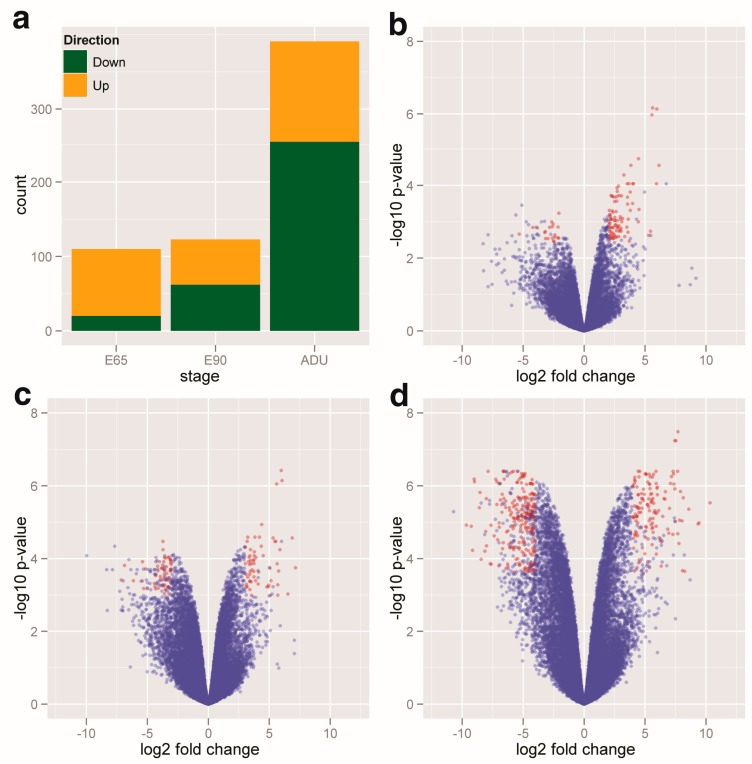
The DEGs at the E65, E90 and ADU time points compared to the E33. Total 484 DEGs were composed by 175 up-regulated genes and 309 down-regulated genes, in which the number of down-regulated genes has an increasing trend from embryonic stage to adult (**a**). The volcano plots show DEGs (red circles) at E65 (**b**) E90 (**c**) and ADU (**d**) time points compared to the E33, respectively.

Myogenesis and hypertrophy are two vital topics in muscle development of pigs, which are turned on by different genes and miRNAs at different time points. Compared to the E33 time point, there were in total 484 DEGs composed of 175 up-regulated genes and 309 down-regulated genes from the E65, E90 to ADU time points (Supplementary S1). Interestingly, a significant increasing trend of down-regulated DEGs from embryonic stage to adult was detected based on their increasing amounts at different time points (Figure 1a). The volcano plots also showed that the up-regulated DEGs quantitatively dominated the embryonic stage (Figure 1b,c), however, it altered to increasing down-regulated DEGs after the postnatal stage determined by numerous genes remarkably down-regulated opposite to the E33 (Figure 1d). The Mfuzz soft clustering also showed that down-regulated trends dominated the gene expression patterns in all clustering structures (Supplementary S1). Meanwhile, the number of DEGs at different time points with an increasing pattern was demonstrated by significant points (red circles), which implied the distinct scenario of gene regulation between the embryonic stages and postnatal adults.

### 2.2. Linear Expression Patterns of DEGs

**Figure 2:**
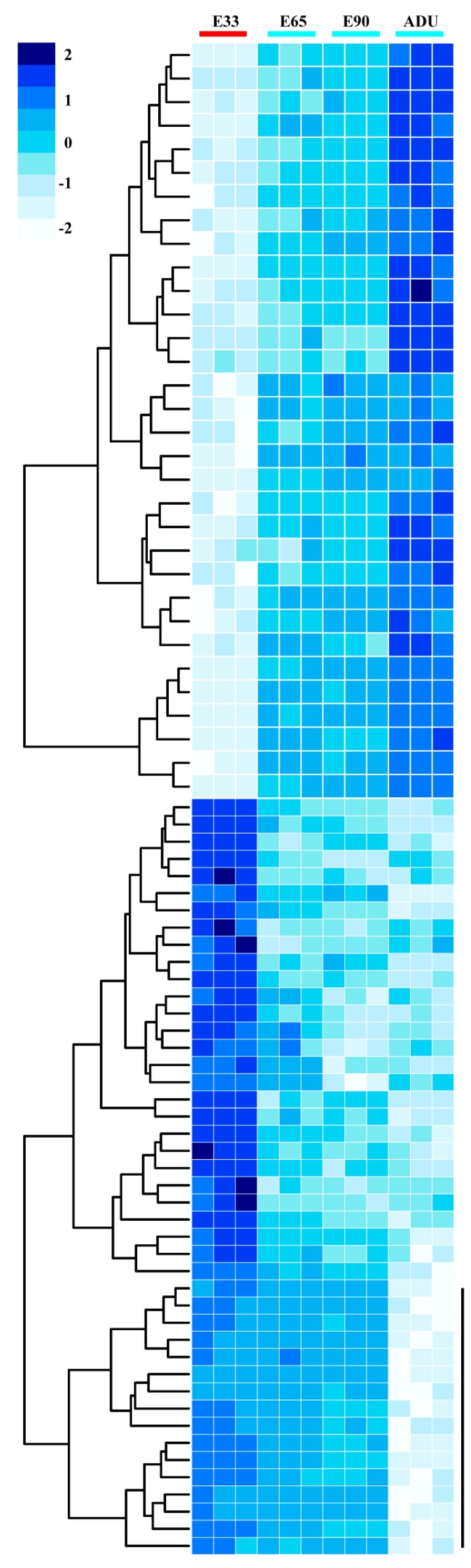
Linear expression patterns of DEGs at 4 time points. Two clusters, including 32 linear increasing DEGs (**up**-panel) and 44 linear decreasing DEGs (**down**-panel), were conducted by complete hierarchical clustering method. Bottom vertical bar indicates the 16 DEGs cluster significantly down-regulated only at ADU time point. All DEGs expression levels were centered to have mean zero and standard deviation one.

The linear expression patterns (LEPs) of DEGs was defined to indicate consensus change in the developmental procedures and showed the sustained roles of involved genes in the whole durations. Two LEP blocks at the four time points were constituted by 32 linear up-regulated DEGs (Figure 2, upper panel) and 44 down-regulated DEGs (Figure 2, lower panel), which were determined by complete hierarchical clustering method. Moreover, 16 DEGs were only extremely down regulated at adult time point (ADU) based on the centered relative expression levels (Figure 2, vertical bar).

### 2.3. Characterization of the miRNA Patterns

**Figure 3:**
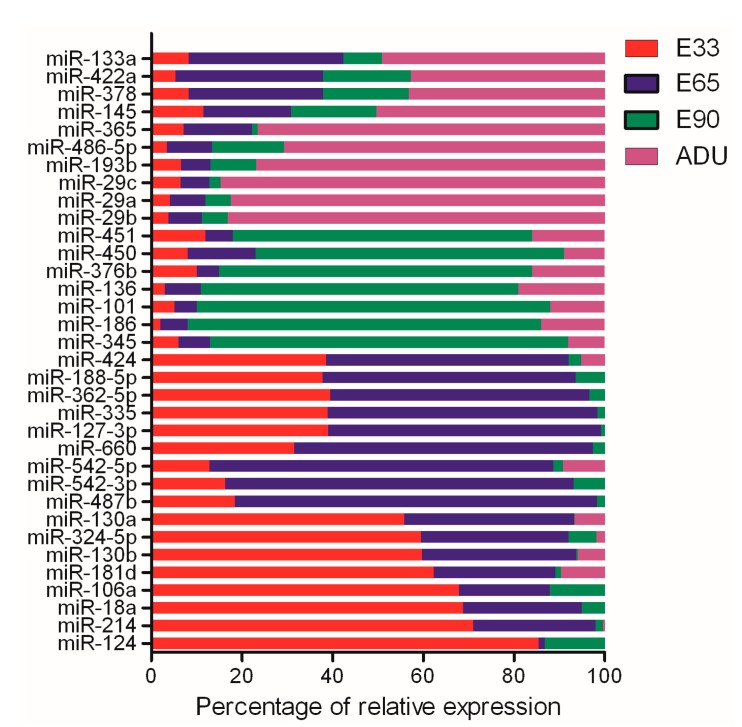
The percentage of relative expression of 34 miRNAs at 4 developmental time points. Three category patterns from the DEMs were clustered based on the expression percentages including early-middle embryonic stage from miR-124 to miR-424, late embryonic stage from miR-345 to miR-451 and adult stage from miR-29b to miR-133a, respectively. The relative expressions were calibrated to same levels on the basis of normal distribution with standard deviations.

As a type of regulator of gene expression at the translational level, miRNAs may involve the pathways of target genes that were differentially expressed at different time points. The expression patterns of differentially expressed miRNAs (DEMs) were determined by the normalized relative percentages at four time points. In total 34 DEMs were significant at different time points, which constituted three category patterns of DEMs for the developmental stages (Figure 3). There were 17 DEMs, represented by *miR-124* and *miR-214*, dominating the miRNA-regulated gene expression in the early metaphase of embryonic stage and 10 DEMs, *miR-29* for instance, were highly expressed at the postnatal stage. Meanwhile, 7 DEMs like *miR-186* and *miR-345* were specifically expressed at the embryonic metaphase stage, which implied the fine distinction in the middle and later period of embryonic stages.

### 2.4. Gene Ontology Annotation (GOA) of DEGs

**Figure 4:**
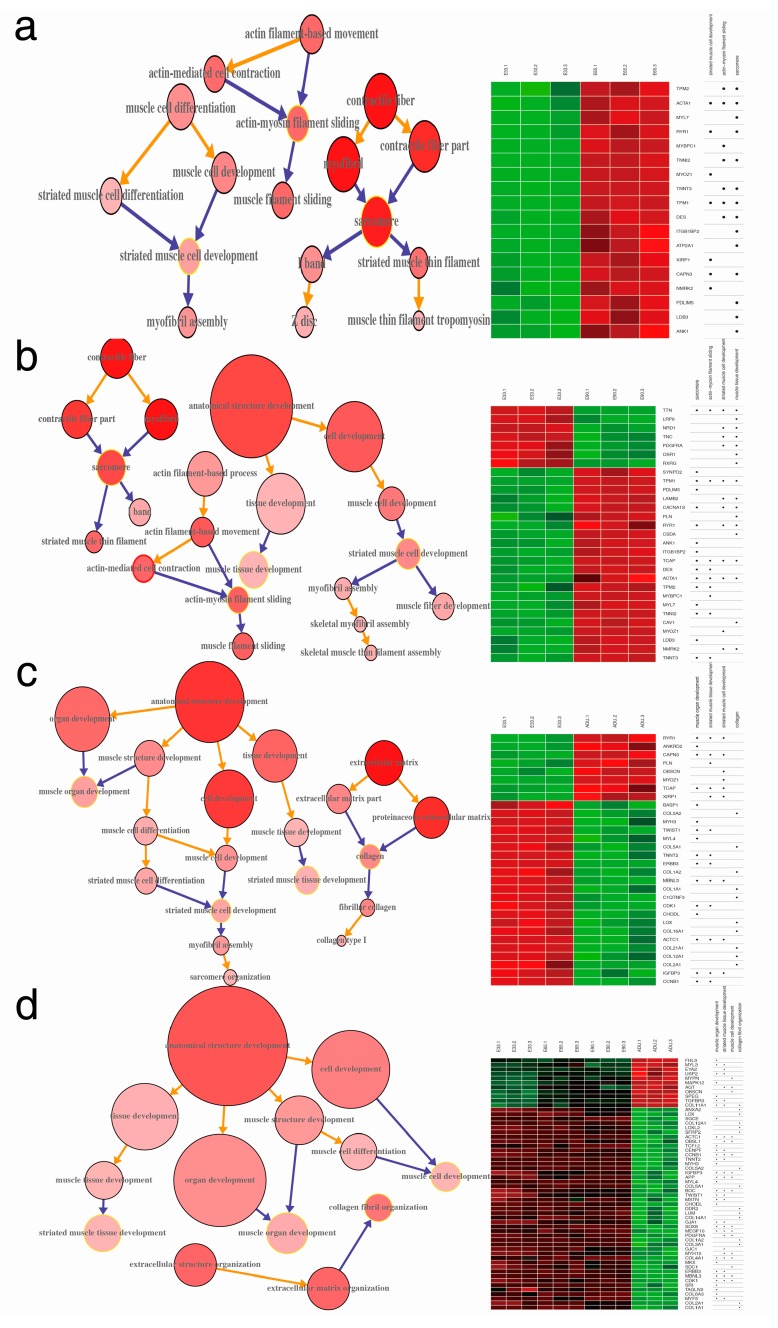
The gene ontology annotation (GOA) of biological events at different time points. Three comparisons with the E33 time point were corresponding to three biological events, microstructure assembly of sarcomere at early embryonic stage (**a**); myofibril formation at late embryonic stage (**b**); and function establishments of myoblast cells after birth (**c**); The overall comparison between embryonic stage and adult stage discriminated the shaping myofibril structure event and muscle functions formation, respectively (**d**). The size of the GO circle corresponded to the number of members of the GO and the color related to the number of DEGs involved in it. Heatmaps in each graph were assembled by the normalized DEGs at four time points.

The GOA database provided the high-quality annotations of genes using the GO standardized vocabulary, which make it possible to understand the biological meaning of the DEGs. GOA analysis at each time points was conducted based on the DEGs compared to the E33 (Supplementary S1). The results showed that the biological events were explicitly generated at the different time points and were embodied in the microstructure assembly of sarcomere at the early time point (E33 and E65), the myofibril formation at the latter embryonic stage (E90) and the function establishments of myoblast cells after postnatal stage (ADU), respectively (Figure 4a, Figure 4b and Figure 4c). Because the total amount of muscle fibers after birth does not change in pigs, the result (Figure 4d) showed that the increasing muscle fibers and basic muscle structure shaping at prenatal period were mainly involved by muscle cell development and fibril organization based on the GO annotation of DEGs. The tissue and organ development of muscle were processed at the postnatal stage. Combined with GOA and the hierarchical clustering, the heatmap results demonstrated that the DEGs clusters served different GOs in each biological event. There were 18 DEGs, TPM2, ACTA1, MYL7 *et al.*, forming 3 GOs including striated muscle cell development, actin-myosin filament sliding and sarcomere to contribute the biological event of sarcomere microstructure assembly (Figure 4, right panel). According to the heatmaps of DEGs, the down-regulation trend was shown based on the number of DEGs along with time points. On the other hand, DEG GOs were connected to biological events to refine the development of skeletal muscle in pigs. Because of the hierarchy of GOs, DEGs were thus clustered by their biological roles.

### 2.5. Mapping Interactome Networks of DEGs

**Figure 5:**
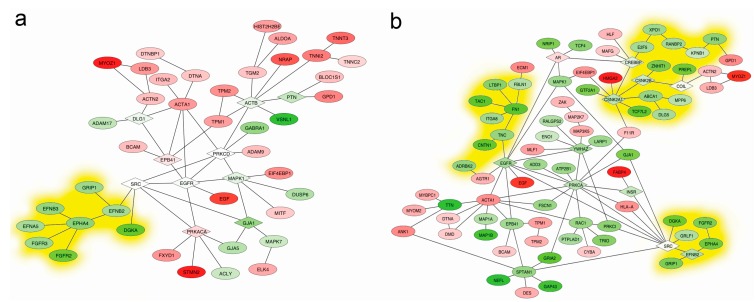

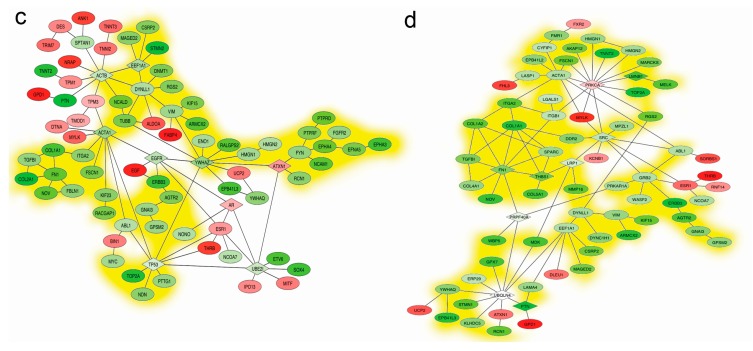
The interactome network mappings for DEGs at different time points. Three comparisons with the E33 time point were corresponding to the early-middle embryonic stage events (**a**); late events (**b**); and adult events (**c**); respectively. The overall comparison between embryonic stage and adult stage was shown during the prenatal and postnatal events (**d**). The down-regulated trend of DEGs was shown as yellow clouds. The color of DEGs indicated up (red) or down (green) regulation. The connective node was represented as a white node. The expression data were normalized to same calibration.

The interactome network especially based on protein-protein interactions (PPIs) was widely used to identify the genes responsible for the roles in a specific biological process, such as a disease. In order to construct the relationship of DEGs at different time points, the interactome networks were mapped according to the pathway designation (Supplementary S1) and the nearest neighborhood of the DEGs at each time point. The results consistently showed that the down-regulated trend (yellow clouds) of DEGs across the whole duration was appeared via the connected DEGs (nodes) and relationships (edges) in skeletal muscle development (Figure 5). Interestingly, the DEGs in the same interaction zone such as *MYOZ1*-*ACTA1* were integrated as sub-network in most case to play a biological role like the microstructure assembly of sarcomere at the early time points (Figure 5a). However, the *TTN* sub-network in the myofibril formation stage and the *SPTAN1* sub-network in the stage of function establishments of myoblast cells were reversely gated by the key node *TTN* and *SPTAN1* DEGs, respectively. These results also demonstrated that the critical node like *SRC* and *PKCA* genes had important roles to construct the interactome networks, although these genes were not in DEGs.

### 2.6. Discussion

Embryonic differentiation and postnatal hypertrophy are two distinct events in skeletal muscle development in pigs, which dominates the commercial value of carcass. Although a family of proteins knows as myogenic regulatory factors (*MRFs*) was considered to regulate muscle differentiation, and also downstream effectors, the target genes, were easy to identify using microarray or sequencing technology, how to dissect the biological meaning and the relationship underneath the DEGs or DEMs is still a challenge in the data mining area [17,18]. There are two strategies to understand the roles of DEGs, one is the GO-based semantic explanation and the other is the gene pathway oriented interpretation. Unfortunately, neither can integrate the whole meaning of the genes, especially to solve the relationship of the involved genes. On the other hand, the GO itself cannot demonstrate the detailed expression pattern at different conditions such as time points.

In this study, we adopted the DEGs-driven concepts towards resolving the biological meaning on the events of myogenesis and hypertrophy of skeletal muscle in pigs, which constructed the GO schemes and the interactome networks of muscle development at different time points. Our results primarily focused on the biological meaning of GO items and the interactome networks of DEGs, which were different from the methods of comparing functions of DEGs themselves. The common points of DEG clusters were meaningful, rather than its individual functions, and connective networks will provide more integrative information based on GOs. According to the previous studies, the myogenesis process of myoblasts at early stage in pigs was triggered by the *MyoD* transcription factor, which comprehensively activated the relevant genes such as *Myostatin*, *Myogenin* and *Myf-5* to regulate the differentiation of myoblast [19,20,21]. However, how the target genes connect, their relationships, and where the key node is located still remain unknown. Because of the biological meaning-oriented methods, our results could answer these questions just in the matter of GOs and interactome networks. For instance, the sarcomere structure related genes *MYL7*, *MYOZ1*, *TNNTs*, *TPMs* and *MYBPC1 etc.*,whose meaning were categorized into three GOs, sarcomere, actin-myosin filament sliding and striated muscle cell development, by using GOA in this study, were highly differentially expressed at the E65 time point compared to the E33. Further, the details of GOs involved were decomposed into myofibril assembly and more imperceptible structure such as Z-disk based on the DEGs in the GOs. All the explanation about the GOs pointed to the biological event *i.e.*, fine structure assembly of sarcomere. Meanwhile, these DEGs also been submitted to construct the interactome networks at this time point. Based on the knowledge of the interactome database, we explicitly know that the sarcomere assembly event including *MYO1* was controlled by the genes such as *EPB41*, *PRKCD* and *EGFR*, because of the roles of their connectivity and even they were not DEGs at this time point. Many previous reports also indicated that *EGFR* gene had a lot of influence on the physiological processes and pathological changes of muscles involved in the functional maintenance of muscle, tumorigenesis and cancer metastasis [22]. Interestingly, although the *EGFR* gene was not detected by microarray in this study, our results actually still found it as an important node to connect the DEG clusters in the E65 time point by using the interactome database and show its central role in regulating related DEGs of sarcomere assembly events. In addition, the key nodes of biological events at different time points were easily identified in the interactome maps, which were indicated by the centralized nodes like *SPTAN1*, *ATXN1*, *UBE21*, *SRC* and *PRKC etc.* These visualized maps and nodes incorporated with the expression levels were self-explanatory elements to clarify the biological events in the development of skeletal muscle in pigs. Also, the relationships among DEGs, GOs and interactome maps integrated together could provide potential clues to analyze the mechanisms dominating the biological events such as myogenesis in pigs.

We also investigated miRNA profiling at different time points in this study. As another regulatory component, miRNA participates in the regulation of embryonic myogenesis and postnatal hypertrophy of skeletal muscle in pigs. Our results were consistent with previous studies, which demonstrated *miR-29* targeting to the collagen family members such as *COL4A1*, *COL1A2* and *COL1A1* at ADU time points, *miR-148* to the *MITF* and *EIF4BP2* genes at early embryonic stage, and *miR-487* to the *IRS1* gene at the middle stage [23,24]. However, there is a substantial difference in the regulation mechanisms between the miRNAs and the transcripts. The miRNAs contributed to suppress the target genes at post-transcription levels, which may not change the expression level of protein. It may result in the absence of miRNAs in interactome maps.

As a matter of fact, the analysis of GOA and interactome mapping can be used in many scenarios like RNA-Seq, microarray, protein iTraq and even mass spectrum data if only to supply the relative expression data at different conditions. These items in the database will extend the present data to solve the potential biological events and also connect the data to each other to understand the relationship of the targets in the assays.

It is a powerful tool to elaborate the myogenesis and hypertrophy mechanisms of skeletal muscle at four different time points in pigs by integrating the GOs and interactome data. The results shed light on the relationships of the genes related to the development of skeletal muscle in pigs.

## 3. Materials and Methods

### 3.1. Sample Collection and RNA Isolation

According to previous studies [12,25] and the time of fiber type formation, four time points, prenatal period composed of embryonic stage 33 days (E33), 65 days (E65) and 90 days (E90) and adult stage at 185 days after birth (ADU) of Landrace pigs, were chosen to survey the expression of genes and miRNAs. Three replicate fetuses for each time point were collected from three independent sows. Adult pigs were also collected from three independent sows. The longissimus dorsi (LD) muscles of three independent fetal or adult pigs were isolated according to the ethics laws (HBAC20091138) at each time point. The muscle samples were quick frozen in liquid nitrogen and stored at −80 °C for later use. Total RNA was isolated from LD muscle using Trizol reagent (Invitrogen, Carlsbad, CA, USA) followed manufacturer’s instructions. Mixing equal amount of RNAs from three independent samples generated 1 μg RNA pool for each time point. The quality of total RNAs was determined according to the RNA integrity number (RIN > 10) and rRNA ratio (28s/18s > 2) by using Agilent 2100 Bioanalyzer and eukaryote total RNA Nano chips (Agilent Technologies Inc., Santa Clara, CA, USA).

### 3.2. Porcine Gene Chip and Customized miRNA Array

The gene expression profiling at four time points was conducted using GeneChip porcine genome array (Affymetrix, Santa Clara, CA, USA) with corresponding services (CapitalBio Corp., Beijing, China) followed manufacturer’s procedures. Generally, the porcine gene chip contains 23,937 probe sets that interrogate approximately 23,256 transcripts from 20,201 porcine genes. In order to detect the expression level of porcine miRNAs, a new miRNA array contained 1551 porcine miRNA probes including 988 miRNA for human, 350 for rat, 627 for mouse and 546 for pig was designed according to the conservation of miRNAs among mammalian kingdom. The detailed information of the miRNA array was as noted in a previous study [16]. In total, 5 μg pooled RNA was submitted to the hybridization of gene chip and miRNA array.

### 3.3. Statistical Analysis of Gene Chip and miRNA Array

The intensity of probes in gene chips and miRNA arrays was converted to the expression levels by using limma package in R and Bioconductor software [26,27]. The normalization methods were respectively conducted using MAS5 and RMA algorithms in the limma package [28,29]. The original expression levels were fitted to the linear model after removing all controls. DEGs and DEMs between two time points, the E65 *vs.* the E33, were obtained by using standard *t*-test with adjusted *p*-value for multiple comparisons, which was controlled under 0.05 false discovery rate (FDR) using Benjamini-Hochberg method.

### 3.4. Databases and Sequences

Pig genome Sscrofa10.2 was retrieved from Ensembl website [30] and the porcine transcript sequences were from Ensembl BioMart retrieve interface. The homologous gene, its annotation and BLAST tools were downloaded from NCBI website [31]. The mature or hairpin miRNAs were obtained from miRBase database [32]. The miRDeep2 software and its dependency packages including bowtie, ViennaRNA, squid and ranfold were downloaded at their websites [33]. The mammalian conserved miRNA targets and sites were calculated using TargetScan script based on the TargetScan database [34]. Gene ontology search was on the Gene Ontology Consortium [35] and AmiGO website [36].

### 3.5. Gene Ontology Annotation (GOA)

The GOA of genes was functionally annotated based on GO database using AmiGO tools. The annotation enrichment analysis, functional clustering, KEGG pathway mapping and homologue match were utilized the DAVID tools [37,38]. The enrichment analysis for gene ontology was carried out using topGO package. The orthologs genes in human for Sscrofa10.2 were annotated by using Ensembl BioMart interface. Homologue miRNAs were annotated on the basis of miRNA family entries in miRBase. The heatmaps of DEGs within GOA were scaled up to normal distribution with standard deviation.

### 3.6. Interactome Mapping of the DEGs

The protein-protein interaction (PPI) network (PIN) was constructed by using CCSB interactome database, and the DEGs were homologous converted to human genes based on Ensembl BioMart homologs database before inputting to the networks. Note that the pre-defined PIN in human may lose some corresponding nodes in pigs because there is not one-to-one mapping in two species and these nodes will be removed before constructing maps. The R packages, GeneAnswers and BioNet, were used to construct the interaction maps among genes, and Cytoscape software was used to refine the maps [39,40,41]. The size and color of nodes and relationships in PIN were standardized on the basis of the normalization expression levels of DEGs.

## 4. Conclusions

Our results demonstrated that there were in total 484 DEGs and 34 DEMs detected in the period from embryonic stage to adult in pigs, which consisted of two LEPs with consensus changes. Moreover, myogenesis events, microstructure assembly of sarcomere and myofibril formation of muscle fibers, were presented based on the GOA analysis, resulting in the interaction networks of DEGs. The interactome mapping may provide extra clues and widen our search for more candidates for further research on the relationship between DEGs and interactions, as well as shedding light on genes and gene networks involved in influencing the growth and development of porcine skeletal muscle.

## Supplementary Materials

Supplementary materials can be found at http://www.mdpi.com/1422-0067/15/12/21788/s1.
